# The effects of genetic manipulation, dieldrin treatment and irradiation on the mating competitiveness of male *Anopheles arabiensis* in field cages

**DOI:** 10.1186/1475-2875-13-318

**Published:** 2014-08-13

**Authors:** Hanano Yamada, Marc JB Vreysen, Jeremie RL Gilles, Givemore Munhenga, David D Damiens

**Affiliations:** Insect Pest Control Laboratory, Joint FAO/IAEA Division of Nuclear Techniques in Food and Agriculture, International Atomic Energy Agency, Vienna, Austria; Department of Life Sciences, University of the West Indies, St Augustine, Trinidad; Center for Opportunistic, Tropical and Hospital Infections, National Institute for Communicable Diseases, Sandringham, Johannesburg, South Africa; Wits Research Institute for Malaria, School of Pathology, Faculty of Health Sciences, University of Witwatersrand, Johannesburg, South Africa

**Keywords:** Sterile insect technique, Male quality, ANO IPCL1, *Rdl*, Fried index

## Abstract

**Background:**

To enable the release of only sterile male *Anopheles arabiensis* mosquitoes for the sterile insect technique, the genetic background of a wild-type strain was modified to create a genetic sexing strain ANO IPCL1 that was based on a dieldrin resistance mutation. Secondly, the eggs of ANO IPCL1 require treatment with dieldrin to allow complete elimination of female L1 larvae from the production line. Finally, male mosquito pupae need to be treated with an irradiation dose of 75 Gy for sterilization. The effects of these treatments on the competitiveness of male *An. arabiensis* were studied.

**Methods:**

The competitiveness of ANO IPCL1 males that were treated either with irradiation or both dieldrin and irradiation, was compared with that of the wild-type strain (*An. arabiensis* Dongola) at a 1:1 ratio in 5.36 m^3^ semi-field cages located in a climate-controlled greenhouse. In addition, three irradiated: untreated male ratios were tested in semi-field cages (1:1, 5:1 and 10:1) and their competition for virgin wild-type females was assessed.

**Results:**

The ANO IPCL1 males were equally competitive as the wild-type males in this semi-field setting. The ANO IPCL1 males irradiated at 75 Gy were approximately half as competitive as the unirradiated wild-type males. ANO IPCL1 males that had been treated with dieldrin as eggs, and irradiated with 75 Gy as pupae were slightly more competitive than males that were only irradiated. Ratios of 1:1, 5:1 and 10:1 irradiated ANO IPCL1 males: untreated wild-type males resulted in 31, 66 and 81% induced sterility in the female cage population, respectively.

**Conclusions:**

An irradiation dose of 75 Gy reduced the competitiveness of male ANO IPCL1 significantly and will need to be compensated by releasing higher numbers of sterile males in the field. However, the dieldrin treatment used to eliminate females appears to have an unexpected radioprotectant effect, however the mechanism is not understood. A sterile to wild-type ratio of 10:1 effectively reduced the population’s fertility under the experimental field cage conditions, but further studies in the field will be needed to confirm the efficiency of sterile ANO IPCL1 males when competing against wild males for wild females.

## Background

The sterile insect technique (SIT) is an environmentally friendly, species-specific biological control tactic for the management of selected insect pests, that requires the mass production and sequential release of large numbers of sterile insects into a target population [1]. The male insects are exposed to ionizing radiation for sexual sterilization, and compete with wild males to mate with females in the field, thereby inducing sterility in the native female population, which will result in a decline in the target population in subsequent generations.

The successful implementation of SIT against the malaria vector *Anopheles arabiensis* relies primarily on the sterile males’ competitiveness and mating success in the field. The competitiveness of colony reared, sterilized, and released male mosquitoes is linked to numerous biological parameters, such as longevity, flight performance, spatial occupation of the habitat, available sperm complement, and mating behaviour [2–5]. Each of these parameters could be influenced by the various steps of the production process of the sterile males, three of which in particular (in addition to the rearing process and laboratory colonization [6]) may induce a significant decline in overall quality of the *An. arabiensis* males. First, to eliminate potentially disease-transmitting female mosquitoes from field release material, a genetic sexing strain (GSS) for *An. arabiensis* (ANO IPCL1) based on a dieldrin-resistant mutation was previously developed [7]. The presence of the dieldrin resistance gene (*Rdl*) and/or the complexity of the chromosomal translocation induced in this strain could lead to inherent biological differences between the ANO IPCL1 males and the wild-type males. Second, during the sexing procedure at the egg stage, embryos are exposed to dieldrin and the resistant males survive while the susceptible females die. The ability to eliminate females at this early developmental stage has advantages, such as reduced production costs, labour and space requirements [7], but may impact the biology or performance of the treated adults. Finally, the male pupae are subjected to a dose of 75 Gy of gamma or X-ray irradiation resulting in >98% sterility [8] before they emerge and are ready for release.

The GSS ANO IPCL1 has been evaluated in terms of reliability of female elimination [7], rearing parameters [9], strain management [7], and radiation sensitivity [7, 8]. GSSs based on dieldrin resistance have been produced in the past for *An. arabiensis* [10] and *Anopheles gambiae* [11], but these strains no longer exist. Life history traits such as developmental parameters of the immature stages, adult size (wing length), adult fecundity and longevity were similar between the ANO IPCL1 and the wild-type *An. arabiensis* Dongola strain with the exception of the high intrinsic sterility observed in the ANO IPCL1 [9]. Moreover, the ANO IPCL1 strain had a similar sperm production pattern as the Dongola strain, suggesting that this specific gene-translocation did not modify this reproductive parameter [12]. However, it has been observed in other strains, that there may be an intrinsic loss of vigour related to the dieldrin resistance gene [13]. Females of homozygous-resistant *An. gambiae* and *Anopheles stephensi* strains were less responsive to oviposition stimuli, produced fewer eggs per unit of blood, were less mobile when seeking hosts or oviposition sites, and responded slower to simulated predators as compared to females of heterozygous-resistant and homozygous-susceptible strains [13, 14]. The resistant males were generally less successful in competing for females, which might be related to their lower mating success because their reaction to female mating cues (as to predator movements) was generally slower. These results should be considered carefully as there was no attempt to distinguish strain from resistance gene effects. However, in the light of other findings, the general fitness and quality of ANO IPCL1 must be scrutinized with a series of experiments to ensure that there are not prohibitive reductions in competitiveness.

Dieldrin (C_12_H_8_Cl_6_O), a potent insecticide, is a very persistent organic pollutant known to be absorbed and stored mostly in the adipose tissue of insects and mammals [15, 16]. Fourth instar larvae of the GSS ANO IPCL1 can be treated in 0.1 ppm dieldrin solutions to eliminate females, which can also be achieved at the embryonic stage by treating <12-hour old eggs in 3–4 ppm dieldrin solutions. Dieldrin adheres strongly to many surfaces, and is absorbed through the chorion of the mosquito eggs, where its residues are retained by the mosquito until adulthood [17]. Furthermore, significant differences were seen in adult male longevity after treating the eggs with the insecticide compared to untreated males (Yamada, unpublished data). Dieldrin treatment however had a positive impact on sperm production in adult males that were irradiated as pupae with the production of sperm continuing in the first week of adult life, while males that had only been irradiated as pupae without the dieldrin treatment did not produce new sperm cells during adult life [12]. It was therefore hypothesized that dieldrin treatment might have a radioprotectant effect on the *An. arabiensis* germinal cells.

Earlier studies documented the effect of irradiation on some *Anopheles* male life history traits, i.e., irradiating pupae of *An. arabiensis* with gamma rays (with doses ranging from 25–100 Gy) had little impact on pupal survival, adult longevity and fecundity of females mated with irradiated males [18]. Sterilizing male mosquitoes at the adult and pupal stages using X-rays (at doses ranging from 35–105 Gy) had some negative effects on adult longevity, possibly due to the stress imposed on them while in the irradiation canister, as well as somatic damage caused by irradiation [8]. Moreover, X-ray irradiation at 70 Gy decreased the number of sperm in testis in two-day old males and inhibited subsequent sperm production during adult life [12].

The current study aimed to evaluate the mating competitiveness of male ANO IPCL1 compared to wild-type males to assess any potential prohibitive reductions in competitiveness due to the chromosomal aberrations or the *Rdl* gene in the GSS. Second, the effects of irradiation on the mating competitiveness of ANO IPCL1 males compared to fertile wild-type males were evaluated, and finally, the effects of the dieldrin treatments in addition to the irradiation of ANO IPCL1 eggs were assessed on adult male competitiveness when competing against untreated wild-type males. The present study was carried out in support of a feasibility study to assess the potential use of the SIT as part of an area-wide integrated pest management (AW-IPM) strategy against *An. arabiensis* in the Northern State of the Republic of Sudan.

## Methods

The general methodology of the various experiments remained identical but according to the treatment different treated:untreated male ratios were used.

### Origin and rearing conditions of mosquitoes

The *An. arabiensis* GSS ANO IPCL1 was developed in 2008 at the Insect Pest Control Laboratory (IPCL) of the Joint FAO/IAEA Division, Seibersdorf [7]. The ANO IPCL1 and the wild-type *An. arabiensis* Dongola strains were reared in a climate-controlled room maintained at a temperature of 27 ± 1°C and 60 ± 10% relative humidity. The light regime was LD 12:12 hours photoperiod, including dusk (one hour) and dawn (one hour) transitional periods. Larvae were reared in plastic trays (40 × 29 × 8 cm) containing ± 1.5 L of deionized water at a density of approximately 500 first instar larvae (L1) per tray. Larvae were fed a 1% IAEA diet solution using the same feeding regime as described by Damiens *et al*. [19]. Pupae were collected and placed in small plastic cups inside a fresh adult cage for emergence. Adults were kept in standard 30-cm cubic insect cages (Megaview Science Education Services Co Ltd, Taiwan) and continuously supplied with 10% [w/v] sucrose solution with 0.2% methylparaben [20]. Females were blood-fed weekly on thawed, defibrinated bovine blood using a modified Hemotek feeding apparatus (Discovery Workshops, Accrington, UK) [21]. Gravid females were allowed to oviposit in plastic cups with black lining containing a wet sponge over which a filter paper was placed.

### Irradiation dose and procedure

As previous studies on irradiation induced sterility in this strain showed low residual fertility (8 ± 1%) after exposure to 75 Gy of X-rays [8] this dose was used for the following series of experiments. Male pupae aged 24–36 hours were irradiated with a dose of 75 Gy using a Rad source 2400 X-Ray irradiator (Rad Source Technologies Inc, Suwanee, GA, USA). The pupae were placed in stackable plastic plates in a small amount of water as described by Yamada *et al.* [8, 22]. For each independent repetition (there was a total of three repetitions) a total of 1,700 ANO IPCL1 males were irradiated. Controls consisted of 1,700 *An. arabiensis* Dongola (wild-type) males, which did not undergo irradiation. Five-hundred virgin *An. arabiensis* Dongola females were sexed manually under a stereoscope and were kept in separate cages. A dosimetry system based on the Gafchromic® HD-810 film (International Specialty Products, NJ, USA) was used for the calibration of the X-ray irradiator and to measure the dose-time accuracy prior to the study. The environmental parameters during irradiation with the Rad source X-Ray irradiator were 24°C, 50-60% RH. The pupae were placed in standard 30 × 30 × 30-cm rearing cages containing a sugar source for emergence.

### Release into semi-field cages

*Anopheles arabiensis* Dongola males irradiated as pupae displayed a significantly lower level of mating success when tested in larger cages (2.16 m^3^) compared to the standard small (30 cm) rearing cages [23]. In larger cages, the biological impairments caused by the irradiation, such as reductions in flight ability, female recognition, energy reserves, and swarming capacity seems to become more visible [23] and better reflects the actual competitiveness of the insects. Therefore, to test the different treatments, large (5.36 m^3^) semi-field cages (Live Monarch, Boca Raton, FL, USA) were used for each independent repetition and were set up in a climate-controlled greenhouse at 25°C ± 1 and 60% ± 5% RH. Each cage contained a larval-rearing tray half filled with water surrounded by three sugar sources. The trays served as an attractant, which facilitated the localization of the sugar sources by the mosquitoes. The mosquitoes were released from the rearing cages into the semi-field cages. All groups were allowed to mate for a period of two nights. The experimental set-up was repeated independently for a total of three times (i.e., each group had three replicates).

### Collection of females from the semi-field cages

On the second morning after release, females were recollected from the field cages by an aspirator and returned to 30 × 30 × 30-cm rearing cages. Each control and treatment group was placed into their own respective cages. The females were offered a blood meal that afternoon and the following day. Oviposition cups were placed in the cages as described in the ‘Origin and rearing conditions of mosquitoes’ section.

### Experimental design

#### Competitiveness of ANO IPCL1 males

To determine any intrinsic loss of potency as a result of its complex chromosomal aberration and the presence of the *Rdl* gene, the competitiveness of the ANO IPCL1 males was compared to that of *An. arabiensis* Dongola males (competing for virgin *An. arabiensis* females) at a 1:1:1 ratio. The mosquitoes were released from the rearing cages into the semi-field cages at following ratios: a) Control Dongola: 100 *An. arabiensis* Dongola males + 100 virgin *An. arabiensis* Dongola females; b) Control ANO IPCL1: 100 ANO IPCL1 males + 100 virgin *An. arabiensis* Dongola females; and, c) (×4) Treatment 1:1 ratio: 100 ANO IPCL1 males + 100 fertile *An. arabiensis* Dongola males + 100 virgin *An. arabiensis* Dongola females. The set of replicates and controls were completed simultaneously as part of one overall experiment.

### Competitiveness of irradiated ANO IPCL1 males

The mosquitoes were released from the rearing cages into the semi-field cages at following ratios: a) Control fertile: 100 *An. arabiensis* Dongola males + 100 virgin *An. arabiensis* Dongola females; b) Control sterile: 100 irradiated ANO IPCL1 males + 100 virgin *An. arabiensis* Dongola females; c) Treatment 1:1 ratio: 100 irradiated ANO IPCL1 males + 100 fertile *An. arabiensis* Dongola males + 100 virgin *An. arabiensis* Dongola females; d) Treatment 5:1 ratio: 500 irradiated ANO IPCL1 males + 100 fertile *An. arabiensis* Dongola males + 100 virgin *An. arabiensis* Dongola females; and, e) Treatment 10:1 ratio: 1000 irradiated ANO IPCL1 males + 100 fertile *An. arabiensis* Dongola males + 100 virgin *An. arabiensis* Dongola females. The experimental set up was repeated independently for a total of three times (i.e., each group a-e, included three replicates).

### Competitiveness of dieldrin-treated, irradiated ANO IPCL1 males

The experimental set-up was the same as the previous one, but included an additional step in which the eggs of the ANO IPCL1 strain were exposed to dieldrin as part of the sexing process, thereby eliminating all females at the embryonic stage. The method was as follows: to determine the effects of the dieldrin exposure on the ANO IPCL1 eggs, females of ANO IPCL1 were offered a blood meal, and oviposition cups were placed in the cage overnight and removed the following morning (aged ≤ 12 hours). The eggs were concentrated by rinsing them off of the filter paper into plastic cups lined with filter paper, to which they adhere. The eggs were estimated and separated into batches of 2,000-3,000 eggs per exposure tube (made of plastic, 5 cm in diameter, the bottom of which was sealed with fine netting). These tubes allow simple and rapid exposure and rinsing of batches of eggs. The tubes containing the eggs were then placed into 50 ml of 3 ppm dieldrin at a constant temperature of 25°C for two hours. After exposure, the eggs were collected and rinsed before placing them into white cups lined with filter paper containing de-ionized water and 640 μl 1% FAO/IAEA larval diet, [19]. Larvae were allowed to mature to adulthood in standard rearing trays.

The mosquitoes were released from the rearing cages into the semi-field cages at following ratios: a) Control fertile: 100 *An. arabiensis* Dongola males + 100 *An. arabiensis* Dongola virgin females; b) Control sterile: 100 irradiated ANO IPCL1 males + *An. arabiensis* Dongola virgin females; c) Control dieldrin-treated, sterile: 100 treated, irradiated ANO IPCL1 males + 100 *An. arabiensis* Dongola virgin females; d) (2×) Treatment 1: irradiated, 5:1 ratio: 500 irradiated ANO IPCL1 males + 100 fertile *An. arabiensis* Dongola males + 100 virgin *An. arabiensis* Dongola females; and, e) (2×) Treatment 2: dieldrin-treated and irradiated, 5:1 ratio: 500 dieldrin-treated, irradiated ANO IPCL1 males + 100 fertile *An. arabiensis* Dongola males + 100 virgin *An. arabiensis* Dongola females. The experimental set-up was repeated independently for a total of two times (i.e., each group a-c, had two replicates in addition to the same treatments in the previous section *‘Competitiveness of irradiated ANO IPCL1 males’ ,* therefore a-c had a total of five replicates, and d and e had a total of four replicates).

### Parameters recorded and statistical analysis

The number of females recovered from each of the semi-field cages after the two-night mating period was recorded for each treatment group and repetition. Hatch rate (egg fertility) was calculated for each female population (i.e., each individual cage) by dividing the number of L1 larvae by the total number of eggs laid. An average value was calculated for each treatment and variance calculated according to the method described by Hooper and Horton [24]. The competitive index, ‘C’ , defined by Fried [25] was calculated for each cage using hatch rates from the fertile control (Hn), sterile control (Hs) and the treatment cages (Ho) as follows:  where N is the number of ‘normal’ males (untreated) and S is the number of sterile males.

To evaluate the effects of sterile male releases on the cage populations’ resulting fertility, the induced sterility (IS) was calculated as 100% minus the residual fertility value, which was obtained by dividing the observed hatch rate (Ho) by the control hatch rate (Hn). Hatch rates for each test groups were pooled to get an average value per treatment, and were compared by ANOVA. Graphics and statistical analyses were performed using Microsoft Excel 2003 (Microsoft, WA, USA; 1985–2003) and Minitab release 13.32 (Minitab; 2000). In all cases, the alpha level was *P* <0.05.

## Results

For all experiments, the recovery rate of females from the field cages ranged between 85 and 95%.

### Competitiveness of ANO IPCL1 males

The fertility in the Dongola strain control population was 81.3% (measured as percentage of eggs hatched) while that of the untreated ANO IPCL1 control population was 29.3%. At a 1:1 ANO IPCL1 male: wild-type male ratio, the mean fertility of the cage population was 53.8% ± 12.3 giving a competitiveness index of 1 (1.04, Table 1). The untreated ANO IPCL1 males when competing with an equal number of wild-type males were able to induce 35% sterility into the cage population.

**Table 1:** **Insemination rate (in percent) and number of eggs produced from cages containing either a 1:1, 5:1 or 10:1 ratio of irradiated ANO IPCL1 to Dongola males**

	Insemination rate		Number of eggs produced by the cages
Control Dongola	53.6 ± 5.1	ab	784.5 ± 41.7
Control Irr GSS	39.2 ± 2.7	a	747.5 ± 736.1
Irr GSS (1:1)	60.0 ± 2.8	ab	1202 ± 675.1
Irr GSS (5:1)	56.1 ± 11.9	ab	1572.5 ± 3
Irr GSS (10:1)	75.6 ± 3.6	b	2102.5 ± 1

Significantly differences between mean insemination rates are indicated by different letters (p < 0.05).

### Competitiveness of irradiated ANO IPCL1 males

The number of females inseminated and number of eggs collected from the cages control fertile, control sterile, 1:1 ratio, 5:1 ratio and 10:1 ratio are presented in Table 1. Due to the high variability between replicates, there is no significant difference between treatments and controls (ANOVA, F_4,10_ = 0.64, P = 0.64). The number of females inseminated show a significant difference between treatments and controls (ANOVA, F_4,5_ = 9.0, P < 0.05). However, only control sterile and 10:1 ratio are significantly different.

**Figure 1:**
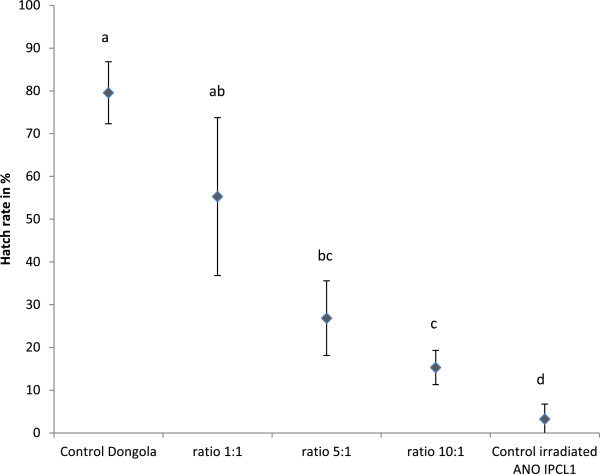
**Mean of hatch rates (±SD) from cages containing either a 1:1, 5:1 or 10:1 ratio of irradiated ANO IPCL1 to Dongola males and from cages containing only irradiated ANO IPCL1 or Dongola males (controls).** Significant differences between mean hatch rate are indicated by different letters (*p* < 0.01).

The mean hatch rates significantly differ between treatments and controls (ANOVA, F_4,10_ = 31.09, P < 0.05) (Figure 1). The mean fertility of the 1:1 (sterile: wild-type) ratio treatment was not significantly different from that of the control fertile and that of 5:1 ratio treatment (Figure 1) because of the high variability of the observed hatch rates between replicates, and of the 5:1 ratio treatment. Moreover, mean fertility of the 5:1 and 10:1 (sterile: wild-type) ratio treatments were not significantly different to each other, but significantly different from the two controls (fertile and sterile). In addition, during the experiment, full sterility was not attained even with the highest sterile: wild-type ratio 10:1 as it was significantly different from the sterile control. The IS in the cage populations reached 30.5, 66 and 81% for the sterile: wild-type 1:1, 5:1 and 10:1 ratios, respectively.

#### Competitive index

The C values for irradiated GSS males competing in the semi-field cages (0.5) were similar irrespective of the ratio tested (Table 2) as the ratio is accounted for in Fried’s formula [25].

**Table 2:** **Variance of competitiveness values for the irradiated GSS at varying ratios (right), and comparison of competitiveness of untreated GSS, irradiated GSS and treated/irradiated GSS (left)**

	CI for untreated GSS, irradiated GSS and dieldrin-treated, irradiated GSS			CI for irradiated GSS at varying ratios		
Statistic	GSS	Irr GSS	Tx IRR GSS	Irr GSS (1:1)	Irr GSS (5:1)	Irr GSS (10:1)
Ratio S/N	1	5	5	1	5	10
Size	4	4	4	3	3	3
Avg Hn ± SE	0.813 ± 0.00	0.751 ± 0.03	0.751 ± 0.02	0.796 ± 0.04	0.796 ± 0.04	0.796 ± 0.04
Avg Hs ± SE	0.293 ± 0.00	0.022 ± 0.00	0.042 ± 0.01	0.032 ± 0.02	0.032 ± 0.02	0.032 ± 0.02
Avg Ho ± SE	0.538 ± 0.06	0.382 ± 0.09	0.204 ± 0.07	0.553 ± 0.11	0.268 ± 0.05	0.153 ± 0.02
IS (%)	35.37	49.13	72.77	30.54	66.28	80.77
Avg C	**1.038**	**0.205**	**0.673**	**0.467**	**0.447**	**0.533**
SE (±)	0.215	0.043	0.128	0.187	0.049	0.042
CV	20.761	20.974	19.053	40.106	11.011	7.878
95% CL (±)	0.686	0.137	0.408	0.806	0.212	0.181
CI (lower)	**0.352**	**0.068**	**0.265**	**-0.339**	**0.235**	**0.352**
CI (upper)	**1.723**	**0.341**	**1.082**	**1.273**	**0.659**	**0.713**

N = number of untreated males; S = number of (semi) sterile males; Hn = egg hatch of the cross fertile Dongola ♂x Dongola ♀; Hs = egg hatch from the cross (semi) sterile GSS ♂ x Dongola ♀; Ho = observed hatch rate; IS = induced sterility; C = competitiveness value; CV = coefficient of variance; CL = confidence limits. Egg hatch is expressed as a rate.

### Competitiveness of dieldrin-treated/irradiated ANO IPCL1 males

**Figure 2:**
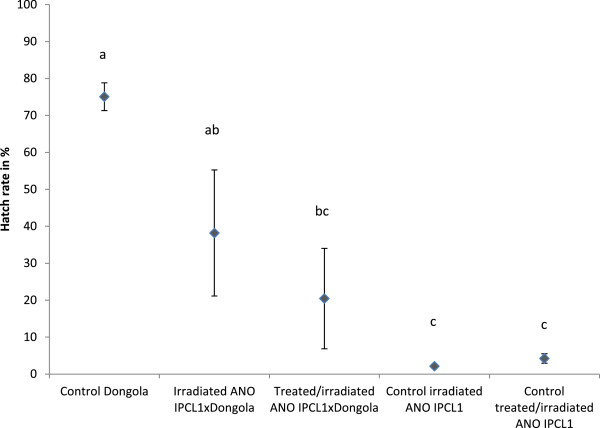
**Mean of hatch rates (±SD) from cages containing either irradiated ANO IPCL1** ***versus*** **Dongola males or treated/irradiated ANO IPCL1** ***versus*** **Dongola at 5:1 ratio and from cages containing only irradiated ANO IPCL1, treated/irradiated ANO IPCL1 or Dongola males (controls).** Significantly difference between mean hatch rate are indicated by different letters (p < 0.01).

The mean hatch rates were significantly different according to the treatment (ANOVA F_4,9_ = 11.59, P < 0.05) (Figure 2). The two treatments irradiated and dieldrin-treated irradiated GSS males showed high variability precluding significant differences between hatch rates. At 5:1 (treated: wild-type) ratio, the IS value for the dieldrin-treated irradiated ANO IPCL1 males was higher (IS = 73%) than that achieved by ANO IPCL1 males which only underwent irradiation (IS = 49%) (Table 2).

#### Competitive index

Although statistically insignificant (since the confidence intervals overlapped), the competitiveness index of dieldrin-treated irradiated ANO IPCL1 was higher than that of only irradiated ANO IPCL1 males (Table 2).

## Discussion

In the context of AW-IPM programmes including an SIT component, it is well known that colonization, mass rearing and irradiation processes could affect the mating competitiveness of the released males [26–31]. Male *An. arabiensis* males destined for release in programmes that include a SIT component, need to endure several treatments: (1) for the purpose of sex separation, a complex translocation of the *Rdl* gene was induced for the development of the genetic sexing strain (GSS) ANO IPCL1 [7]; (2) dieldrin treatments at immature stages are necessary for female elimination from the production line; and, (3) irradiation at pupal (or adult) stage is required for the sterilization of the males prior to release. These treatments could have a negative impact on the mating competitiveness of the males produced.

The results indicate that prohibitive reductions on competitiveness due to biological effects of the presence of the *Rdl* gene, and the complex translocation associated with this GSS can be precluded as the competitiveness of the ANO IPCL males was not compromised when competing with wild-type males for wild-type females in a field-cage setting. Under the experimental conditions of the current study, the ANO IPCL1 males were equally competitive compared to the wild-type strain. Similar findings were documented in the past: the chromosomal aberrations inherent in the MACHO strain of *Anopheles albimanus* had no negative effects on the competitiveness of the males [32], and males of *Culex tarsalis* carrying a chromosomal translocation were also equally competitive as wild-type males when assessed in large laboratory cages (~4 m^3^) [33].

According to the competitiveness index elicited by a comparison of the observed hatch rate with the expected hatch rate in this current study, irradiated ANO IPCL1 males (IRR GSS) exhibited a C value of around 0.5 (where a value of 1.0 suggests equal competitiveness), meaning that the males were approximately half as competitive as wild-type males when evaluated in this given scenario. Assuming that the C value obtained in this field-cage study was comparable to hypothetical values that can be expected in the field, the number of irradiated ANO IPCL1 males released in a control programme against this species would need to be doubled to arrive at an equal level of competitiveness. It needs to be noted that in an operational programme, the operational sterile to wild male ratio required in the field would probably need to be higher as the competitiveness of the sterile males is also influenced by their dispersal capacity, their spatial occupation of the habitat and their survival [34].

Due to the variation of experimental methodologies regarding cage sizes, release ratios and adult densities, the comparison of C values from different studies as presented in the literature is difficult and non-transferable to other situations. For the same species, with the same sex ratio but in different cages, a C value of 0.76 and 0.34 was obtained for *An. arabiensis* Dongola males irradiated at 70 Gy released in small (30 cm) and large (2.16 m^3^) semi-field cages, respectively [23]. In field tests assessing male mating competitiveness for the GSS of *An. albimanus* (MACHO) in El Salvador against newly colonized wild-type males, a C value of 0.785 was reported [32]. A C value between 0.84 and 0.92 was obtained for an artificially infected *Aedes polynesiensis* strain generated by introgressing *Wolbachia* from *Aedes riversi* when evaluated in semi-field cages (2.9 m^3^) and conditions against F1 offspring from wild-caught *A. polynesiensis* females [35]. An *Aedes albopictus* (*Stegomyia albopicta*) line (ARwP), harbouring a new *Wolbachia* infection achieved a C value of 1.0 when they competed with naturally infected males (SR line) in laboratory cages (30 × 30 × 30 cm) and in a greenhouse (120 m^3^) [36]. Unfortunately, many of these reports do not indicate the variance of the C values and it is therefore difficult to deduct if C values between treatments truly differ. The coefficient of variation of the C values reported in this paper is very high making it difficult to make sound conclusions on the actual competitiveness and the differences between groups. The competitiveness index therefore was used as an indicator of mating success, which is influenced by various intrinsic or extrinsic factors and to compare treatment and control groups.

The irradiated ANO IPCL1 males additionally treated with dieldrin at the egg stage were more competitive than those that had only been irradiated. Relevant side effects of dieldrin treatments have also been observed in a study on sperm production in *An. arabiensis* that showed that dieldrin-treated/irradiated males had a higher level of sperm production at day 6 (post emergence) than males that were only irradiated [12]. These data suggest a radio protectant effect of dieldrin, or the dieldrin treatment procedure itself, on the *An. arabiensis* germinal cells. This current study seems to confirm that the somatic damage incurred by irradiation in ANO IPCL 1 males could be slightly reduced by the presence of dieldrin residues, which are known to be retained by the insect to adulthood [17]. Alternatively, the physiological stress of the dieldrin treatment potentially could have enhanced or stimulated the radiation protection mechanisms in the mosquitoes [37]. There are only a few chemicals known to have a radiation-protecting effect in mosquitoes: one of these is dimethyl sulphoxide (DMSO) that supplied to adult *Anopheles atroparvus* decreased the number of dominant lethal mutations induced by the X-rays, but the treatment reduced the longevity of the adult mosquitoes [38]. As observed in most of the competitiveness experiments in large cages, there was a high variability between replicates [39], however such variability was not observed in the controls which excluded cage volume as the source of the variation. Another factor that might have contributed to the variations is the heterogeneous irradiation of the pupae. A variation of -10 and +7% of the central dose value in the stackable plates used for pupal irradiation has been measured in the X-ray irradiator [22], meaning that a dose range of 67.5-80.25 Gy was received by the pupae. The pupae also varied slightly in age, ranging from 24- to 36-hours old. This could have contributed to the proportional variation in the final level of sterility in the males. Even if the utmost is done to avoid any variation in the absorbed irradiation dose, none of the exposed pupae would receive an equal dose, with a different impact on their biological quality as a result. The above is corroborated by another competitiveness study that obtained a high degree of variability in hatch rates of eggs of individual females mated with males irradiated as pupae, suggesting small differences in the irradiation dose received [23]. In any case, increasing the number of replicates would be advantageous though this requires a large amount of mosquitoes of homogeneous age at one time which is difficult to achieve and involves a great deal of work.

According to the irradiated: untreated male release ratios studied, a minimum ratio of 10:1 was required to reduce the cage population’s fertility by 81% and to inseminate 75% of the females present in the cage. Similar levels of induced sterility (81 ± 4%) were obtained in *Aedes albopictus* studies with a ratio of 5:1 irradiated to untreated males using similar semi-field cages [40]. For many other insect species, when deployed in the field as part of integrated pest management (IPM) programmes, it has been necessary to release sterile males in numbers adequate to obtain ratios in the field in favour of sterile males as these proved less competitive than wild males; In Burkina Faso, a sterile to wild male tsetse fly (*Glossina palpalis gambiensis*) ratio of 7:1 to 10:1 was most effective in achieving eradication of the targeted wild population [41]; ratios of 40:1 were the target for the suppression of a codling moth (*Cydia pomonella*) population in British Colombia, Canada [42]; and sterile male Mediterranean fruit flies (*Ceratitis capitata*) were released obtaining a 100:1 sterile: wild male ratio in the field, which resulted in a significant decline in fertility of a wild population in Guatemala [43]. Moreover, the frequency of releases will influence the efficiency of the method and have to be frequent enough to maintain a critical overflooding ratio [44]. A release of *An. albimanus* males in El Salvador proved effective even though sterile males were determined to be two to four times less competitive than their wild counterparts [45].

## Conclusions

It is interesting to note that in these experiments, the level of induced sterility in the female population by the un-irradiated GSS males was nearly as high as the induced sterility achieved by the GSS males irradiated at 75 Gy, while competitive integrity was maintained in the non-irradiated males. This leads to the assumption that due to the high natural sterility and observed equal competitiveness of ANO IPCL1 in the field cage setting, one could increase overall competitiveness of the males by reducing the irradiation dose and thereby ultimately achieving a higher level of sterility in the target population [46]. An additional advantage of this approach is that the sterility will have residual effects as any male progeny resulting from released males mated with wild females will carry forth the same level of natural sterility, producing a somewhat residual, yet self-limiting effect. Nevertheless, these assumptions must be bolstered by a series of field-based assessments before explicit recommendations can be made. The assessment of male *Culex tritaeniorhynchus* carrying a complex chromosomal aberration showed that they were highly competitive when released with laboratory-colonized females in a laboratory setting, but were uncompetitive when released during a field study [47] suggesting that long-term insectary maintenance can select an assortive mating behaviour. This underlines the importance of using wild females to evaluate the competitiveness of altered genotypes for a more accurate indication of the projected field performance of released males.

Moreover, even if the C values obtained here in field cages have to be treated with caution, the results presented here are a good initial indicator for the relative mating competitiveness of ANO IPCL1 males that have undergone different degrees of treatments compared to laboratory-reared wild-type males (*An. arabiensis* Dongola). However they require verification in a true natural setting in the field and further studies on the evaluation of the mating success of treated ANO IPCL1 males with wild females are required before any conclusions can be drawn regarding the suitability of this GSS for SIT operations, or any sound recommendations on required released sterile to wild male ratios can be made.

## References

[1] Knipling EF (1955). Possibilities of insect control or eradication through the use of sexually sterile males. J Econ Entomol.

[2] Oliva CF, Damiens D, Benedict MQ (2014). Male reproductive biology of Aedes mosquitoes. Acta Trop.

[3] Oliva CF, Damiens D, Vreysen MJ, Lemperière G, Gilles JRL (2013). Reproductive strategies of *Aedes albopictus* (Diptera: Culicidae) and implications for the sterile insect technique. PLoS One.

[4] Thornhill R, Alcock J (2001). The evolution of insect mating systems. iUniverse.com,Inc.

[5] Calkins CO, Parker AG, Dyck VA, Hendrichs J, Robinson AS (2005). Sterile insect quality. Sterile Insect Technique. Principles and Practice in Area-Wide Integrated Pest Management.

[6] Oliva CF, Benedict MQ, Lemperiere G, Gilles J (2011). Laboratory selection for an accelerated mosquito sexual development rate. Malar J.

[7] Yamada H, Benedict MQ, Malcolm CA, Oliva CF, Soliban SM, Gilles JRL (2012). Genetic sex separation of the malaria vector, *Anopheles arabiensis*, by exposing eggs to dieldrin. Malar J.

[8] Ndo C, Yamada H, Damiens DD, N’do S, Seballos G, Gilles JR (2013). X-ray sterilization of the *An. arabiensis* genetic sexing strain ‘ANO IPCL1’ at pupal and adult stages. Acta Trop.

[9] Oliva CF, Benedict MQ, Soliban SM, Lemperiere G, Balestrino F, Gilles JR (2012). Comparisons of life-history characteristics of a genetic sexing strain with laboratory strains of *Anopheles arabiensis* (Diptera: Culicidae) from northern Sudan. J Med Entomol.

[10] Lines JD, Curtis CF (1985). Genetic sexing systems in *Anopheles arabiensis* Patton (Diptera: Culicidae). J Econ Entomol.

[11] Davidson G, Hamon J (1962). A case of dominant dieldrin resistance in *Anopheles gambiae* Giles. Nature.

[12] Damiens D, Vreysen MJB, Gilles JRL (2013). *Anopheles arabiensis* sperm production after genetic manipulation, dieldrin treatment, and irradiation. J Med Entomol.

[13] Rowland M (1991). Behaviour and fitness of ãHCH/dieldrin resistant and susceptible female *Anopheles gambiae* and *An.stephensi* mosquitoes in the absence of insecticide. Med Vet Entomol.

[14] Rowland M (1991). Activity and mating competitiveness of yHCH/dieldrin resistant and susceptible male and virgin female *Anopheles gambiae* and *An. stephensi* mosquitoes, with assessment of an insecticide-rotation strategy. Med Vet Entomol.

[15] Nebeker AV, Dunn KD, Griffis WL, Schuytema GS (1994). Effects of dieldrin in food on growth and bioaccumulation in mallard ducklings. Arch Environ Contam Toxicol.

[16] Reinert RE (1972). Accumulation of Dieldrin in an Alga (*Scenedesmus obliquus*), *Daphnia magna*, and the Guppy (*Poecilia reticulata*). J Fisheries Res Board Canada.

[17] Yamada H, Jandric Z, Chhem-Kieth S, Vreysen MJB, Rathor MN, Gilles JRL, Cannavan A (2013). *Anopheles arabiensis* egg treatment with dieldrin for sex separation leaves residues in male adult mosquitoes that can bioaccumulate in goldfish (*Carassius auratus auratus*). Environ Toxicol Chem.

[18] Helinski MEH, Parker AG, Knols BG (2006). Radiation-induced sterility for pupal and adult stages of the malaria mosquito *Anopheles arabiensis*. Malar J.

[19] Damiens D, Benedict MQ, Wille M, Gilles JRL (2012). An inexpensive and effective larval diet for *Anopheles arabiensis* (Diptera: Culicidae): Eat like a horse, a bird or a fish?. J Med Entomol.

[20] Benedict MQ, Hood-Nowotny RC, Howell PI, Wilkins EE (2009). Methylparaben in *Anopheles gambiae s.l.* sugar meals increases longevity and malaria oocyst abundance but is not a preferred diet. J Insect Physiol.

[21] Damiens D, Soliban SM, Balestrino F, Alsir R, Vreysen MJB, Gilles JRL (2013). Different blood and sugar feeding regimes affect the productivity of *Anopheles arabiensis* colonies (Diptera: Culicidae). J Med Entomol.

[22] Yamada H, Parker AG, Oliva CF, Balestrino F, Gilles JRL: **X- Ray induced sterility in*****Aedes albopictus*****.** *J Med Entomol* In press 10.1603/me13223 25118413

[23] Helinski MEH, Knols BGJ (2008). Mating competitiveness of male *Anopheles arabiensis* mosquitoes irradiated with a partially or fully sterilizing dose in small and large laboratory cages. J Med Entomol.

[24] Hooper GHS, Horton IF (1981). Competitiveness of sterilized male insects: a method of calculating the variance of the value derived from competitive mating tests. J Econ Entomol.

[25] Fried M (1971). Determination of sterile-insect competitiveness. J Econ Entomol.

[26] Bloem S, Carpenter JE, Bloem KA, Tomlin L, Taggart S (2004). Effect of rearing strategy and gamma radiation on field competitiveness of mass-reared codling moths (Lepidoptera: Tortricidae). J Econ Entomol.

[27] Andreasen MH, Curtis CF (2005). Optimal life stage for radiation sterilization of *Anopheles* males and their fitness for release. Med Vet Entomol.

[28] El Gazzar LM, Dame DA (1983). Effects of combinations of irradiation and chemosterilization on mating competitiveness of *Culex quinquefasciatus* Say. J Econ Entomol.

[29] Curtis CF (1976). Radiation sterilization.

[30] Sharma VP, Razdan RK, Ansari MA (1978). *Anopheles stephensi*: effect of gamma-radiation and chemosterilants on the fertility and fitness of males for sterile male releases. J Econ Entomol.

[31] Allinghi A, Calcagno G, Petit-Marty N, Gómez Cendra P, Segura D, Vera T, Cladera J, Gramajo C, Willink E, Vilardi JC (2007). Compatibility and competitiveness of a laboratory strain of *Anastrepha fraterculus* (Diptera: Tephritidae) after irradiation treatment. Florida Entomologist.

[32] Kaiser PE, Bailey DL, Lowe RE, Seawright JA, Dame DA (1979). Mating competitiveness of chemosterilized males of a genetic sexing strain of *Anopheles albimanus* in laboratory and field tests. Mosq News.

[33] Ainsley RW, Asman SM, McDonald PT (1978). Laboratory mating competitiveness of *Culex tarsalis* colonies. Proceedings and papers on the Forty-sixth Annual Conference of the California Mosquito and Vector Control Association, Inc.

[34] Vreysen MJB, Dyck VA, Hendrichs J, Robinson AS (2005). Monitoring sterile and wild insects in area-wide integrated pest management programmes. Sterile Insect Technique. Principles and Practice in Area-Wide Integrated Pest Management.

[35] Chambers EW, Hapairai L, Peel BA, Bossin H, Dobson SL (2011). Male mating competitiveness of a *Wolbachia-*introgressed *Aedes polynesiensis* strain under semi-field conditions. PLoS Negl Trop Dis.

[36] Moretti R, Calvitti M (2012). Male mating performance and cytoplasmic incompatibility in a wPip *Wolbachia* trans-infected line of *Aedes albopictus* (*Stegomyia albopicta*). Med Vet Entomol.

[37] Varanda EA, Takahashi CS, Soares AEE, Barreto SAJ (1992). Effect of *Apis mellifera* bee venom and gamma radiation on bone marrow cells of wistar rats treated *in vivo*. Rev Brasil Genet.

[38] Lecis AR, Orru G (1974). Effeti radioprotettivi e tossicita del DMSO su maschi adulti panirradiati di *Anopheles maculippenis artroparvus* (Diptera: Nematocera). Riv Biol.

[39] Bellini R, Balestrino F, Medici A, Gentile G, Veronesi R, Carrieri M (2013). Mating competitiveness of *Aedes albopictus* radio-sterilized males in large enclosures exposed to natural conditions. J Med Entomol.

[40] Madakacherry O, Lees RS, Gilles JRL (2014). *Aedes albopictus* (Skuse) males in laboratory and semi-field cages: release ratios and mating competitiveness. Acta Trop.

[41] Sow A, Sidibe I, Bengaly Z, Bance AZ, Germain J, Sawadogo GJ, Solano P, Vreysen MJB, Lancelot R, Bouyer J (2012). Irradiated male tsetse from a 40-year-old colony are still competitive in a riparian forest in Burkina Faso. PLoS One.

[42] Bloem KA, Bloem S, Tan KH (2000). SIT for codling moth eradication in British Columbia, Canada. Area-Wide Control of Fruit Flies and other Insect Pests.

[43] Rendon P, McInnis D, Lance D, Stewart J (2004). Medfly (Diptera: Tephritidae) genetic sexing: large-scale field comparison of males-only and bisexual sterile fly releases in Guatemala. J Econ Entomol.

[44] Hendrichs JP, Vreysen MJB, Enkerlin WR, Cayol JP, Dyck VA, Hendrichs J, Robinson AS (2005). Strategic options in using sterile insects for area-wide integrated pest management. Sterile Insect Technique. Principles and Practice in Area-Wide Integrated Pest Management.

[45] Weidhaas DE, Breeland SG, Lofgren CS, Dame DA, Kaiser R (1974). Release of chemosterilized males for the control of *Anopheles albimanus* in El Salvador IV. Dynamics of test populations. Am J Trop Med Hyg.

[46] Franz G, Tan K-H (2000). The “Combi fly concept” revisited: How much radiation is required to sterilise males of a genetic sexing strain?. Area-wide Control of Fruit Flies and other Insect Pests.

[47] Baker RH, Reisen WK, Sakai RK, Hayes CG, Aslamkhan M, Saifuddin UT, Mahmood F, Perveen A, Javed S (1979). Field assessment of mating competitiveness of male *Culex tritaeniorhynchus* carrying a complex chromosomal aberration. Ann Entomol Soc Am.

